# Ribavirin Inhibits Parrot Bornavirus 4 Replication in Cell Culture

**DOI:** 10.1371/journal.pone.0134080

**Published:** 2015-07-29

**Authors:** Jeffrey M. B. Musser, J. Jill Heatley, Anastasia V. Koinis, Paulette F. Suchodolski, Jianhua Guo, Paulina Escandon, Ian R. Tizard

**Affiliations:** 1 Schubot Exotic Bird Health Center, College of Veterinary Medicine, Texas A&M University, College Station, Texas, United States of America; 2 Department of Veterinary Pathobiology, College of Veterinary Medicine, Texas A&M University, College Station, Texas, United States of America; 3 Zoological Medicine, Department of Small Animal Clinical Sciences, College of Veterinary Medicine, Texas A&M University, College Station, Texas, United States of America; 4 Morris Animal Foundation Veterinary Student Scholar, College of Veterinary Medicine, Texas A&M University, College Station, Texas, United States of America; George Mason University, UNITED STATES

## Abstract

Parrot bornavirus 4 is an etiological agent of proventricular dilatation disease, a fatal neurologic and gastrointestinal disease of psittacines and other birds. We tested the ability of ribavirin, an antiviral nucleoside analog with antiviral activity against a range of RNA and DNA viruses, to inhibit parrot bornavirus 4 replication in duck embryonic fibroblast cells. Two analytical methods that evaluate different products of viral replication, indirect immunocytochemistry for viral specific nucleoprotein and qRT-PCR for viral specific phosphoprotein gene mRNA, were used. Ribavirin at concentrations between 2.5 and 25 μg/mL inhibited parrot bornavirus 4 replication, decreasing viral mRNA and viral protein load, in infected duck embryonic fibroblast cells. The addition of guanosine diminished the antiviral activity of ribavirin suggesting that one possible mechanism of action against parrot bornavirus 4 may likely be through inosine monophosphate dehydrogenase inhibition. This study demonstrates parrot bornavirus 4 susceptibility to ribavirin in cell culture.

## Introduction

Parrot bornavirus 4 (PaBV-4) is an enveloped, non-segmented, negative sense RNA virus in the family *Bornaviridae*. Recent phylogenetic analysis of viruses within the family *Bornaviridae*, which includes the formerly named Avian Bornavirus, resulted in new nomenclature and taxonomic reorganization of *Bornaviridae* into 5 species: *Mammalian 1 bornavirus*, *Psittaciform 1 bornavirus*, *Passeriform 1 bornavirus*, *Passeriform 2 bornavirus*, and *Waterbird 1 bornavirus* [1]. Epidemiological and experimental results support that PaBV-1, -2, -3, -4, -7, all members of the species *Psittaciform 1 bornavirus*, are etiological agents of proventricular dilatation disease (PDD), a fatal neurologic and gastrointestinal syndrome of psittacines and other birds [2–4]. In infected birds, the virus has a tropism for the brain and nervous tissue of psittacines [5–10], but can be found in a majority of tissues including kidney, medullary cords of the adrenal gland, heart, spleen, liver, lungs, pancreas, testes and ovary [7–10]. Bornaviruses are noncytopathic in cell culture [4,9].

Presently, there is no proven efficacious treatment in birds against PaBV infections or for reducing viral shedding. INF-α inhibits virus infection and viral load of the virus in quail cell culture [11]. Non-steroidal anti-inflammatory drugs, to inhibit or reduce the inflammatory reaction, are currently the principal treatment for PDD [12]. Celecoxid, meloxicam, and cyclosporine are used clinically and experimentally with mixed results [12,13]. These drugs are mainly symptomatic treatments of clinical PDD and their use may be contraindicated. In cockatiels experimentally infected with PaBV-4 and treated with meloxicam, severe weight loss and depression was noted and upon necropsy and testing, there was pathological evidence of PDD and increased PaBV-4 dissemination [14].

Ribavirin (1-ß-D-ribofuranosyl- 1,2,4-Triazole-3-carboxamide), a broad-spectrum antiviral nucleoside analog [15], has *in vitro* and *in vivo* antiviral activity against a broad range of RNA and DNA viruses. In human clinical practice, ribavirin is used, alone or in combination with other drugs, in the treatment of infections with hepatitis C virus, respiratory syncytial virus, and Lassa fever virus [16–21]. Experimentally in veterinary species, ribavirin shows antiviral activity against foot-and-mouth disease virus [22], infectious salmon anemia virus [23], viral hemorrhagic septicemia virus [24], and canine parainfluenza virus [25]. In mice, combination treatment with ribavirin and baicalein provides better protection against influenza virus as compared to the individual chemicals alone [26]. With specific regard to the family *Bornaviridae*, ribavirin, at concentrations ranging from 1 to 10 μg/mL, inhibits the transcription of Borna disease virus 1 (BoDV-1; species *Mammalian 1 bornavirus*) in persistently infected MDCK cells [27]. In neural cell lines infected with BoDV-1, viral titers and viral transcripts decline when infected cells are treated with 20 μM ribavirin (4.9 μg/mL) [28].

Since BoDV-1 is prototypical of the family *Bornaviridae*, to which PaBV-4 is a member, the current research was undertaken to evaluate *in vitro* antiviral effects of ribavirin and the combination of ribavirin with baicalein on PaBV-4 in infected duck embryonic fibroblast (DEF) cells and to evaluate a potential mechanism of action. Our results demonstrated that ribavirin decreased viral protein and mRNA expression in PaBV-4 infected cell cultures. The mechanism of action was likely to include inhibition of inosine monophosphate dehydrogenase (IMPDH) resulting in the reduction of intracellular guanosine nucleotide pools (GMP, GDP, and GTP), and that minimal synergism with the addition of baicalein was demonstrated.

## Materials and Methods

### Media and cells

Duck embryonic fibroblast cells were obtained from stock cells previously grown for the study by Gray et al [4]. To ascertain that the cells were not contaminated with any virus of the species *Psittaciform 1 bornavirus*, an aliquot of the stored DEF cells was assayed by RT-PCR [29]. Throughout this study, DEF were grown in Dulbecco’s Modified Eagle Medium (DMEM) (Life Technologies Co., Grand Island, NY, USA) supplemented with 1% penicillin-streptomycin, (10,000 U/mL—10,000 μg/mL) (Life Technologies Co.) and 5 or 10% bovine calf serum (DMEM5% or DMEM10%, respectively) at 37°C in an atmosphere of 5% CO2.

For experiments, DEF cells were seeded into 24-well cell culture plates (BD, Franklin Lakes, NJ, USA) and grown for 24 to 48 hours until cell layers were 70 to 80% confluent before infection with virus.

### Antiviral compounds

Powdered ribavirin, ≥ 98% pure, was obtained from Sigma-Aldrich (St Louis, MO, USA) and ribavirin capsules, 200 mg, were obtained from Sandoz Inc. (Princeton, NJ, USA). Ribavirin solutions were made by dissolving ribavirin in DMEM with 2% bovine calf serum (DMEM2%). Powdered, ≥ 98% pure, ribavirin was used in the cytotoxicity assay and in ribavirin inhibition study 3; the contents of the ribavirin capsules were used in the cytotoxicity assay and in the other studies except ribavirin inhibition study 3. Baicalein (Sigma-Aldrich) was dissolved in PBS (Sigma-Aldrich) and DMEM5%.

### Avian bornavirus

Parrot bornavirus 4 was obtained from a stock supply of virus that had been cultured and isolated from experimentally infected Patagonia conures (*Cyanoliseus patagonis*) with clinical PDD [4]. The virus was passaged in primary DEF cells maintained in DMEM5%. After 5 days of incubation, virus infected DEF cells were harvested, freeze/thawed three times, and the media-virus suspension was divided into 0.5 mL virus stock aliquots and stored at -80°C.

The virus stock was confirmed to be PaBV-4 by RNA extraction and RT-PCR analysis followed by sequence analysis of the PCR product, as described previously [4,29,30]. Serial 10-fold dilutions of three stock virus aliquots assayed by indirect immunocytochemistry estimated the mean (±SD) infectious titers at 12775 (±2467) ffu/mL.

### Cytotoxicity assay

Cytotoxic effect to DEF cells of ribavirin, baicalein, and guanosine (Sigma-Aldrich) was determined using the 3-(4,5-dimethylthiazol-2-yl)-2,5-diphenyltetrazolium bromide (MTT) assay (Vybrant MTT Cell Proliferation Assay Kit, Invitrogen, Eugene OR). DEF cells were grown in 96-well microplates (BD Falcon, BD) for 24 hours. Thereafter, the medium was replaced with 0.15 mL of DMEM10% containing either ribavirin (≥ 98% pure; concentrations ranging from 0.8 to 2000 μg/mL), the contents of the ribavirin capsules (0.8 to 2000 μg /mL), baicalein (0.01 to 50 μg /mL) or guanosine (1.5 to 140 μg /mL). DMEM10% without supplements, PBS, and 0.85 M Triton X (Sigma-Aldrich) in PBS served as controls. The cells were incubated for a further 4 days before MTT assay was performed according to manufacturer’s recommendation. In brief, the media was removed and 100 μL of fresh DMEM10% was added to each well. From a stock solution of MTT (5 mg/mL), 10 μL was added to each well and incubated at 37°C in an atmosphere of 5% CO2 for 4 hours. From a stock solution of sodium dodecyl sulfate (1 g in 10 mL 0.01 M HCl), 100 μL was added to each well, followed by an additional 10 hours incubation at 37°C in an atmosphere of 5% CO2. Optical density at 570 nm was measured on each well using a microplate spectrophotometer (Fluostar Omega, BMG Labtech, Ortenberg Germany) and computer software program (Omega 1.30, BMG Labtech).

For each of the drugs, two independent experiments, each with 8 replicates per concentration, were performed. Results are report as a percent viability of untreated controls: DEF cells incubated in DMEM10%.

### Ribavirin inhibition studies

In study 1, the potential of ribavirin to inhibit PaBV-4 in infected DEF cells was examined. DEF cells grown in 24-well plates were infected with 0.5 mL of either no PaBV-4 (negative control) or with virus at 128, 13, 1.3, 0.1, and 0.01 ffu/mL. The infected cells were incubated a further 24 hours. Then, ribavirin solution, from ribavirin 200 mg capsules, was added, resulting in final ribavirin concentrations of 0, 0.25, 2.5, or 25 μg/mL. The cells were incubated for a further 5 days, and then assayed by indirect immunocytochemistry. Three independent test runs with 2 replicates for each virus/ribavirin combination were tested.

In study 2, DEF cells were infected with 0.5 mL of control (no virus) or 128 and 13 ffu/mL PaBV-4 stock. Following incubation for 24 hours, ribavirin solutions, made from ribavirin 200 mg capsules, were added resulting in final concentrations of 0, 2.5, 10, and 25 μg/mL ribavirin. After 5 days, the medium was replaced with DMEM10% without ribavirin. Cells were incubated a further 5 days in the absence of ribavirin before analysis by indirect immunocytochemistry. Four independent test runs with 2 replicates per each virus/ribavirin combination were tested. Effective concentration 50% (EC_50_) and 90% (EC_90_) were estimated for cells infected with 64 ffu PaBV-4 per well by fitting the data to a four-parameter logistic model to generate a dose-response curve using SigmaPlot version 10.0.1 (Systat Software, Inc., San Jose, CA, USA).

In study 3, DEF cells grown in 24-well plates were infected with 0.5 mL of 13 or 128 ffu/mL virus stock and incubated for 24 hours. Ribavirin solutions made from ≥ 98% pure ribavirin were added to achieve final concentrations of 0, 2.5, 10.0, or 25.0 μg/mL ribavirin. After 5 days of incubation, the media was removed and replaced with 1.0 mL of DMEM5% without ribavirin. Cells were incubated for a further 0, 10, 14, and 21 days and then harvested for analysis by qRT-PCR. Each virus/ribavirin/time combination was repeated on 3 independent experiments and performed in duplicate.

In study 4, DEF cells grown in 24-well plates were infected with 0.5 mL of 128 or 13 ffu/mL PaBV-4 stock. Following incubation for 24 hours, ribavirin solutions made from ribavirin 200 mg capsules were added, resulting in a final concentration of 0 or 25 μg/mL ribavirin. After one or two days of incubation, the medium in a series of ribavirin treated wells was replaced with 1.0 mL of DMEM10% without ribavirin. In a third series of wells the medium containing ribavirin remained on the cells for five days. Six days after the initial infection with PaBV-4, the plates were analyzed by an indirect immunocytochemistry assay. The experiment was repeated in 2 independent trails with duplicates for each virus/ribavirin/time sample.

### Ribavirin-Guanosine inhibition study

DEF cells grown in 24-well plates were infected with 0.5 mL of 128 ffu/mL PaBV-4 stock and then incubated for 24 hours. Thereafter, combinations of ribavirin and guanosine were added to give a final concentration of 0, 10 or 25 μg/mL and 0, 3, 14 or 28 μg/mL, respectively. The cells were incubated for a further 5 days before being assayed. Four independent trials with duplicates of each ribavirin-guanosine combination were assayed by qRT-PCR and three independent trials with duplicates of each ribavirin-guanosine combination were assayed by indirect immunocytochemistry.

### Ribavirin-Baicalein synergism study

DEF cells grown in 24-well plates were infected with 0.5 mL of 128 or 13 ffu/mL PaBV-4 stock, incubated for 24 hours, then treated with 0.05 μg/mL baicalein and 5.0 μg/mL ribavirin individually or in combination for 5 days, and then assayed by qRT-PCR. Two independent experiments with duplicates were run.

### qRT-PCR

After the removal of the media from the DEF cell cultures, 300 μL of PBS was added to each sample well and stored frozen at -80°C until assayed. Total viral RNA was isolated from 100 μL of the PaBV-4 infected cell / PBS solution using the QIAamp viral RNA mini kit (Qiagen, Valencia, Ca). Purified RNA was eluted in 60 μL elution buffer and stored at -80°C until use.

PaBV-4 phosphoprotein (P) gene mRNA was quantified using Taqman RRT-PCR assay performed with TaqMan Fast Virus 1-Step Master Mix (Invitrogen,Carlsbad, Ca) and PaBV phosphoprotein primers: 5’- AAGAAGAA[Y]CC[Y]TCCATG ATCTC-3’ and 5’-AA[Y]TGCCGAAT[B]A[R]GTCATC- 3’, and Taqman probe 5’-FAM-TCGATAACTG [Y]TCCCTTCCGGTC-BHQ-3’. Each reaction was carried out using 6 μl of total RNA, 0.25 μM primers and 0.25 μM probe in a 12 μl final reaction volume. All reactions were carried out using an ABI 7900HT, and cycling parameters were as follows: 5 min reverse transcription (RT) step at 50°C, RT inactivation and polymerase activation for 1 min at 95°C, and 45 amplification cycles at 95°C for 3 sec and 58°C for 30 sec. All samples were run in duplicate and Taqman RRT-PCR assay was repeated twice. Samples were considered negative when ≥ 37 C_T_.

In separate reactions cellular 18S rRNA was quantified using TaqMan Fast Virus 1-Step Master Mix and Eukaryotic 18S rRNA FAM Endogenous Control (Invitrogen,Carlsbad, Ca) according to the manufactures instructions. Reactions were carried out as described above in an ABI 7900HT.

Analysis of qRT-PCR data was through the relative change in P gene mRNA expression, which was calculated using the 2 ^- ΔΔCt^ method [31,32], where ΔΔC_T_ = (C_T_ P gene (treated)–C_T_ 18s rRNA (treated))—(C_T_ P gene (untreated)–C_T_ 18s rRNA (untreated)).

### Indirect immunocytochemistry assay

Indirect immunocytochemistry assay, to detect PaBV-4 nucleoprotein (N protein), was conducted similar to a previously described method [4]. Briefly, the DEF cells were washed twice for 5 min each time in 0.02 M PBS, fixed for 10 min in 2% paraformaldehyde in 0.02 M PBS, and then washed twice for 5 min each time in 0.02 M PBS. Cells were permeabilized using 1% Triton X-100/0.02 M PBS for 10 min and then washed 3 times for 5 min each time in 0.03% Tween/0.02 M PBS. Blocking was performed for 2 hours in 5% dried milk/0.03% Tween/0.02 M PBS. The primary antibody, chicken IgG anti-PaBV-4 N protein, at a 1:500 dilution in 1% dried milk/0.03% Tween/0.02 M PBS, was added to the cells and then incubated in a humidified chamber for 30 min at 37°C.

Cells were washed 3 times for 5 min each time in 0.03% Tween/0.02 M PBS. Secondary antibody, horseradish peroxidase conjugated rabbit anti-chicken IgG (Sigma-Aldrich) at a 1:500 dilution in 1% dried milk/0.03% Tween/0.02M PBS, was added to the cells and incubated in a humidified chamber for 30 min at 37°C. Cells were washed 3 times for 5 min each time in 0.03% Tween/0.02M PBS and then rinsed in distilled water.

### Digital image analysis to determine numbers of PaBV-4 positive cell foci and area of infected cells

Digital images of 24 well plates stained by an indirect immunocytochemistry assay were made using computerized software (VueScan 9, Hamrick Software, http://www.hamrick.com/) and saved as uncompressed 8-bit grey-scale TIFF files.

The 8-bit grey-scale images were imported into an image analysis software program (ImageJ, National Institutes of Health; http://rsb.info.nih.gov/ij/) [33]. Contrast of each plate image was maximized using the auto brightness/contrast tool. Then using the threshold tool, black and white contrast enhancement of each image was done using the brightness control bar, with visual comparison being made to the original grey-scale images to ensure optimum visualization of positively stained cell foci. The image was then converted to a binary image, with infected cells being black, and saved as a TIFF file.

**Fig 1:**
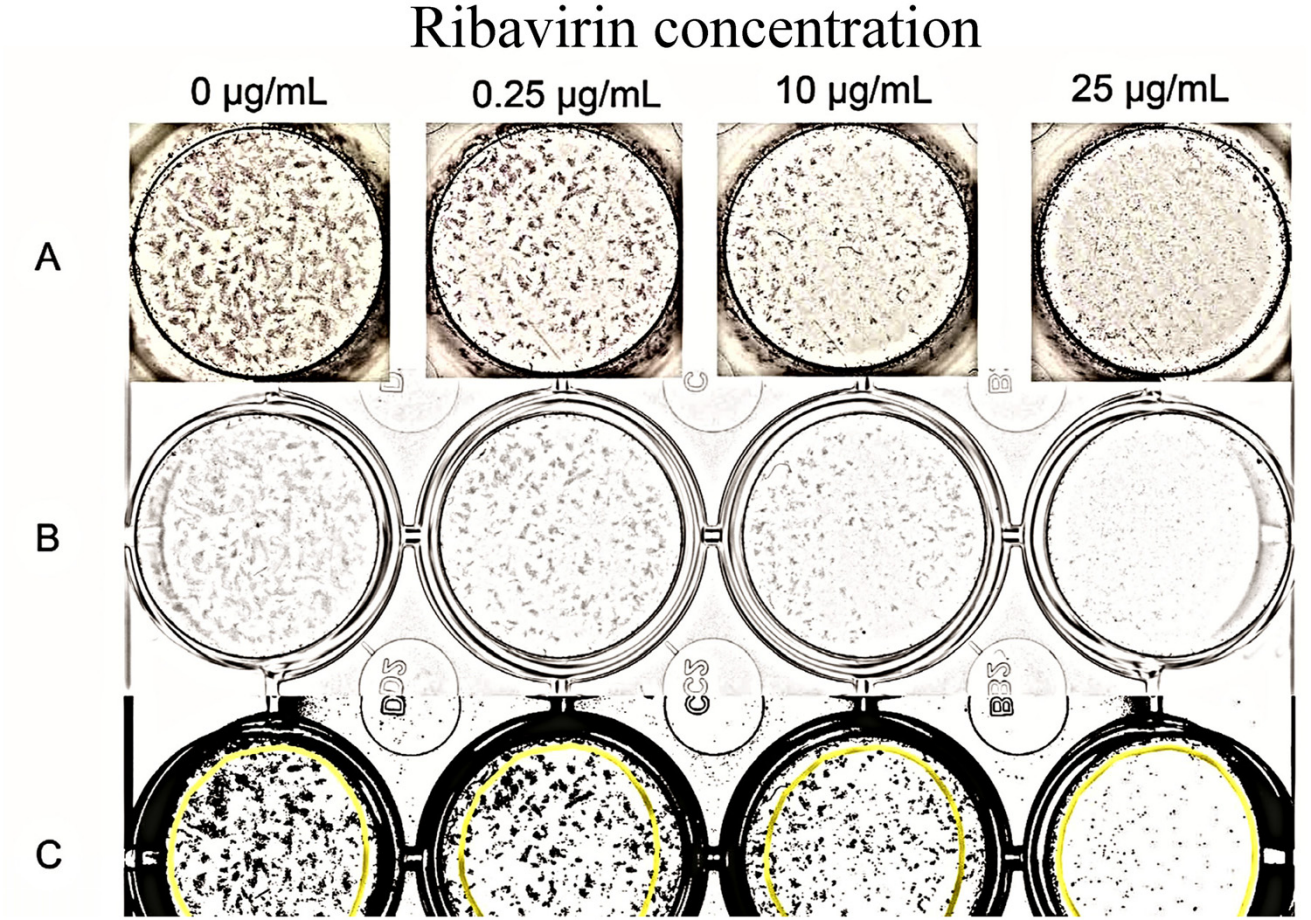
Digital image analysis to determine stained PaBV-4 positive cell foci and area of infected cells. (A) Photograph after indirect immunocytochemistry of wells containing infected duck embryonic fibroblasts infected with PaBV-4 and incubated with or without ribavirin. PaBV-4 nucleoprotein detected as dense shades of grey. (B) Digital 8-bit grey-scale images of the same wells after digital scanning. (C) The same wells after 8-bit grey scale images were enhanced and converted to a binary image for analyses using ImageJ Analyze. The areas of N protein immunocomplex are in black. The area of interest that was analyzed is the same for each well and is within the superimposed yellow circles.

An equally sized area of interest, maximized to include as much of the well as possible, was delineated in each well. The number of PaBv-4 positive cell foci per area of interest was counted using the ImageJ Analyze Particles tool, with particle size gated between 0.0001 to infinity square inch. The area of infected cells, measured as the percent area of black pixels per area of interest, was determined using the macro function Calculate_Black_to_White_Ratio. A representation of the wells and images is provided in Fig 1.

### Statistical analysis

Descriptive statistics of mean ± SD, with standard deviation calculated after 2^-ΔΔCt^ transformation [32], were used to describe relative expression of mRNA. Statistical analysis of qRT-PCR data was performed on mean ΔCt for the independent experimental repeats using One Way Analysis of Variance (ANVOA) with Holm-Sidak method for pairwise comparisons (study 3 and Ribavirin-Guanosine inhibition study) [32]. Descriptive statistics of median, 25^th^ and 75^th^ percentile were used to describe indirect immunocytochemistry results. Statistical analysis was performed using ANOVA test on ranks with Tukey test for pairwise comparisons (study 2). A P ≤ 0.05 was considered statistically significant. SigmaPlot version 10.0.1 was used for performing all statistical analyses (Systat Software, Inc., San Jose, CA).

## Results

### Cytotoxicity of Ribavirin, Baicalein, and Guanosine

**Fig 2:**
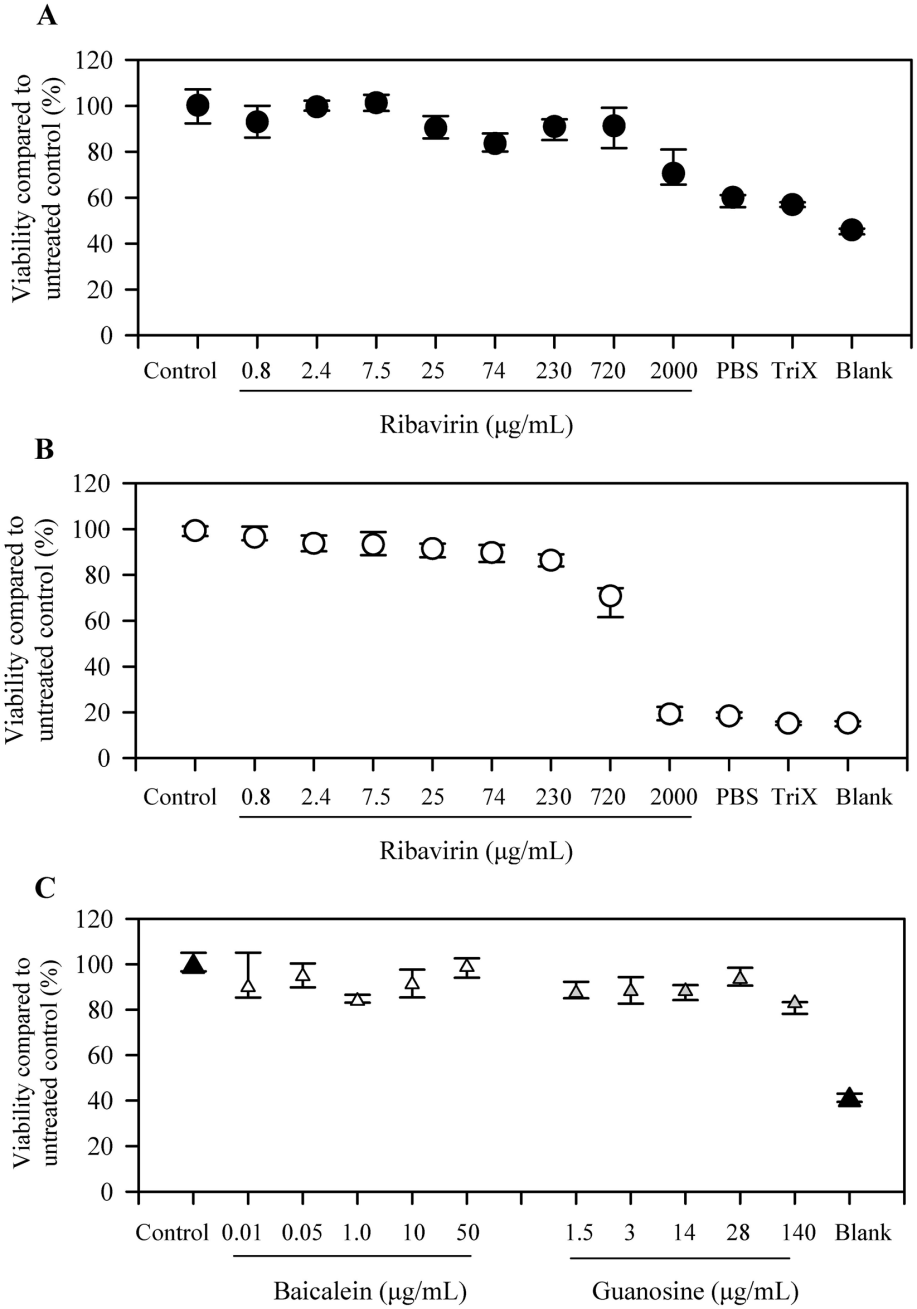
Cytotoxicity on duck embryonic fibroblasts. (A) Cytotoxicity following incubation with HPLC grade ribavirin (> 98% pure). (B) Cytotoxicity following incubation with ribavirin capsule contents. (C) Cytotoxicity following incubation with baicalein or guanosine. Data is depicted as median ± 25^th^ and 75^th^ percentiles.

In order to examine for potential confounding factors of the antiviral agents and other compounds on DEF cell viability, an MTT assay was used to examine the cytotoxicity of the compounds on DEF cells. Cytotoxicity was not determined to be a confounding factor at the concentrations of ribavirin, baicalein, or guanosine used for the virus inhibition experiments in this study. When incubated with ≥98% pure ribavirin (Fig 2A) or contents of ribavirin 200 mg capsule (Fig 2B), cell viability for DEF cells was similar to control values across ribavirin concentrations used in the virus inhibition experiments. When DEF cells were incubated with baicalein or guanosine (Fig 2C), cell viability remained constant across the range of concentrations used in the virus inhibition experiments.

### Ribavirin inhibition of PaBV-4

#### Study 1

This study was performed to determine appropriate doses for PaBV-4 infection and ribavirin treatment. Sufficient numbers of PaBV-4-infected cells were detected only if DEF cells were infected with at least 7 ffu (Table 1). PaBV-4 positive cell foci numbers and area of infected cells decreased with the addition of 2.5 and 25.0 μg/mL ribavirin (Table 1).

**Table 1:** Reduction in viral load of infected duck embryonic fibroblast (DEF) cells following treatment with Ribavirin.

		PaBV-4 positive cell foci		Area of infected cells (%)	
Virus (ffu)	Ribavirin (μg/mL)	Median	(25^th^– 75^th^ percentile)	Median	(25^th^– 75^th^ percentile)
64	0	36	35–37	47.7	47.2–48.2
	0.25	40	33–46	49.5	46.7–52.3
	2.5	73	72–73	30.6	29.0–32.2
	25.0	1	0.5–1.5	0.4	0.4–0.5
7	0	50	48–51	9.1	8.5–9.8
	0.25	73	72–73	12.5	12.0–13.0
	2.5	19	18–20	4.3	4.1–4.5
	25.0	2	2–2	0.5	0.4–0.5
1	0	7	6–7	1.6	1.5–1.6
	0.25	7	6–9	1.3	1.0–1.6
	2.5	4	3–4	0.9	0.9–0.9
	25.0	1	0–1	0.6	0.6–0.6
No virus (negative control)	0	0	0	0.2	0.2–0.3
	0.25	1	0.5–1.5	0.2	0.2–0.2
	2.5	0	0	0.4	0.4–0.4
	25.0	1	0.5–1.5	0.7	0.6–0.8

Cells infected with 0.05 and 0.005 ffu had PaBV-4 positive cell foci and percent area of infected cells similar to No virus (negative control).

#### Study 2

**Fig 3:**
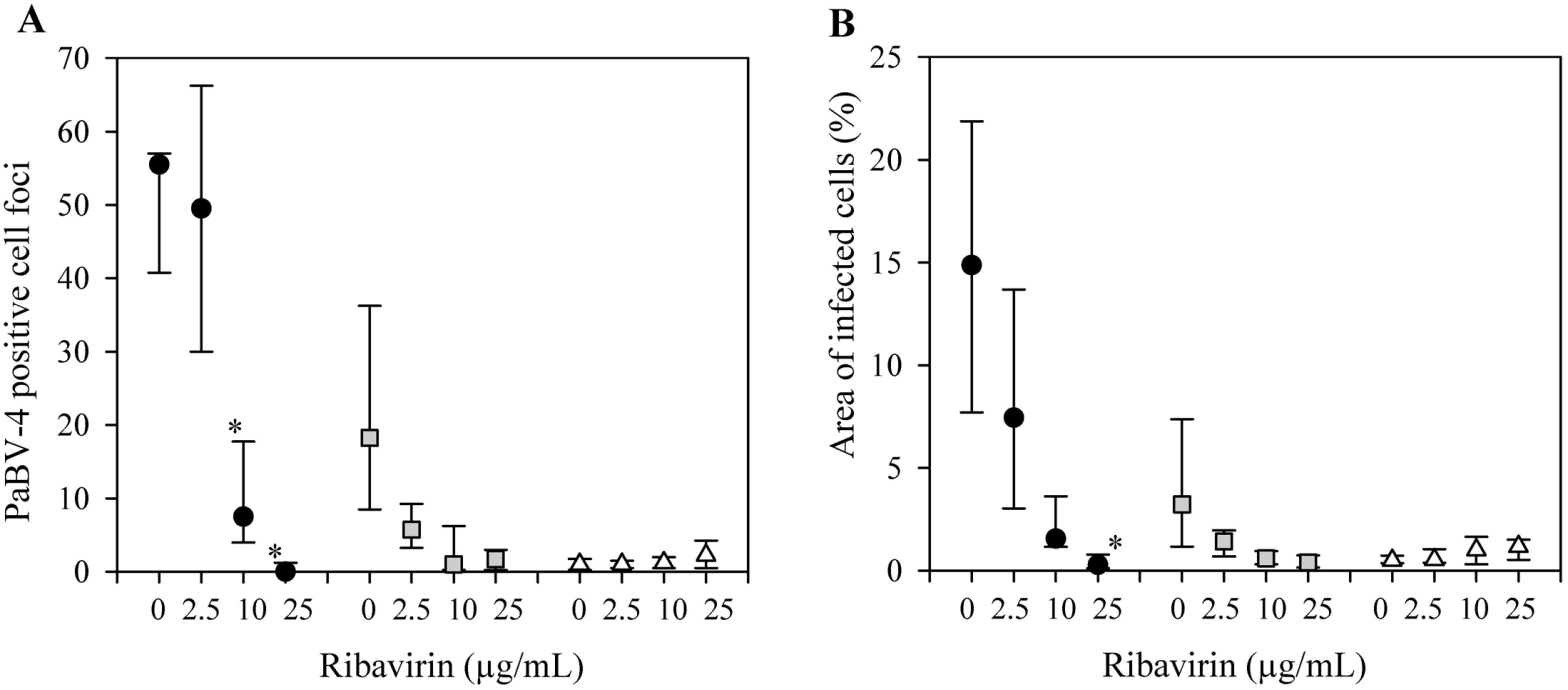
Anti-viral activity of Ribavirin on PaBV-4 in duck embryonic fibroblasts (DEF). (A) PaBV-4 positive cell foci, assessed by indirect immunocytochemistry assay. (B) Percent area of infected cells, assessed by indirect immunocytochemistry assay. DEF infected with 64 ffu (black circle), 7 ffu of PaBV-4 (grey square), or no virus (white triangle), incubated with ribavirin for 5 days, and then incubated a further 5 days in the absence of ribavirin. Data presents as median ± 25^th^ and 75^th^ percentile. *, compared with no added ribavirin (*P* <0.05, ANOVA on ranks with Tukey test).

To determine the antiviral activity of differing concentrations of ribavirin, PaBV-4 infected DEF cells were incubated with increasing concentrations of ribavirin for 5 days, followed by 5 days incubation with DMEM10% without ribavirin. In cells infected with 64 ffu PaBV-4, PaBV-4 positive cell foci were significantly (P<0.05) reduced when treated with 10 and 25 μg/mL ribavirin as compared to untreated control, 0, 8, and 56 positive cell foci, respectively. PaBV-4 positive cell foci decreased in a dose dependent manner in cells infected with 7 ffu, 18, 1, 2 positive cell foci when treated with 0, 10, and 25 μg/mL ribavirin (Fig 3A). A similar dose-dependent reduction in percent area of infected cells was observed (Fig 3B).

For the DEF cells infected with 64 ffu PaBV-4, the estimated EC_50_ and EC_90_, using positive staining foci numbers, were 4.7 and 15.6 μg/mL ribavirin, respectively, and using area of infected cells, were 0.5 and 19.7 μg/mL ribavirin, respectively.

#### Study 3

**Fig 4:**
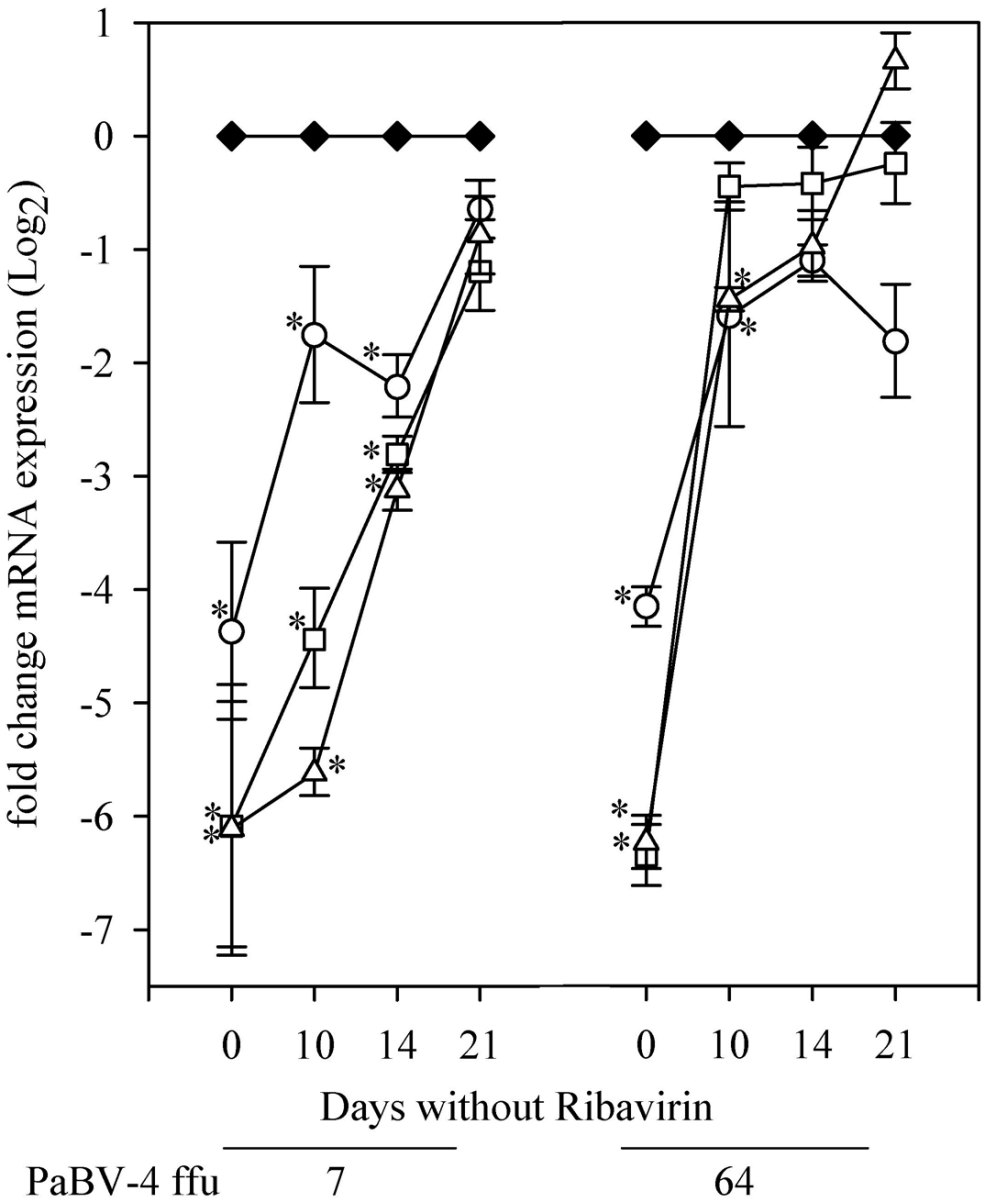
Post-exposure effect of Ribavirin on PaBV-4 P gene mRNA expression. Duck embryonic fibroblasts infected with 7 or 64 ffu PaBV-4, incubated with 0.0 (black diamond), 2.5 (open circle), 10.0 (open square), or 25.0 (open triangle) μg/mL ribavirin. Cells analyzed by qRT-PCR with results (mean ±SD) shown as the fold change in P gene mRNA expression relative to the within day expression. *, compared with no added ribavirin within day (*P* <0.05, ANOVA with Holm-Sidak method).

We investigated the expression of P gene mRNA and duration of time needed for expression to rebound to levels of untreated cells following the removal of ribavirin. After 5 days of treatment with 2.5 to 25 μg/mL ribavirin, expression of P gene mRNA was significantly (P≤ 0.05) less than control of with no ribavirin treatment, ranging from a 4 to 6 fold difference in expression (Fig 4). With the removal of ribavirin from the cells, P gene mRNA expression rose in a time-dependent manner over 21 days, but remained significantly (P≤ 0.05) reduced for up to 14 days after removal of ribavirin.

#### Study 4

**Fig 5:**
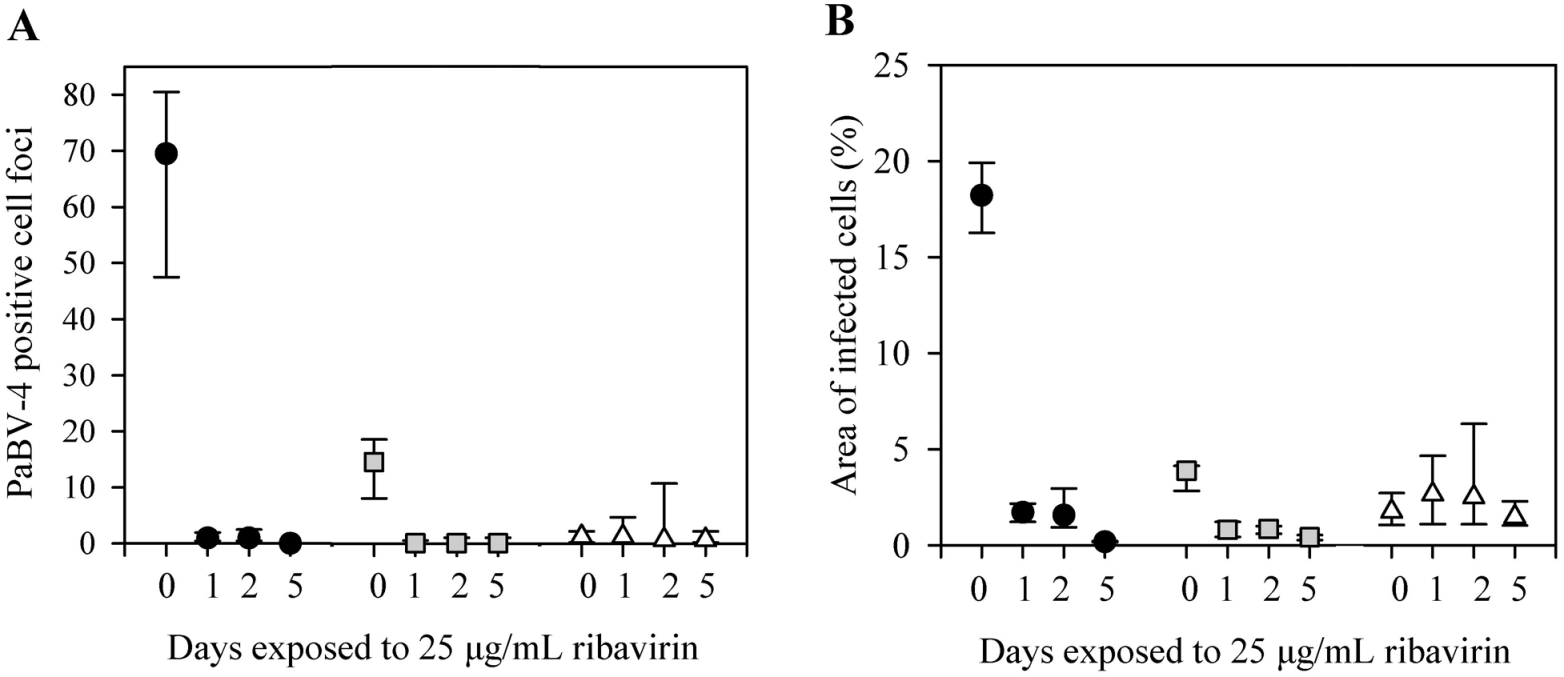
Time dependency to reduce viral load. (A) PaBV-4 positive cell foci, assessed by indirect immunocytochemistry assay. (B) Percent area of infected cells, assessed by indirect immunocytochemistry assay. DEF infected with 64 ffu (black circle), 7 ffu of PaBV-4 (grey square), or no virus (white triangle), incubated for 0, 1, 2, or 5 days with 25μg/mL ribavirin. Data presents as median ± 25^th^ and 75^th^ percentile.

We investigated treatment duration time needed to decrease the viral load of PaBV-4 infected cells. After only 24 hours of exposure to 25 μg/mL ribavirin, followed by 4 days of incubation without ribavirin, PaBV-4 positive foci numbers and percent area of infected cells were less than in the untreated controls with virus (Fig 5).

### Guanosine attenuation of anti-ABV activity of Ribavirin

**Fig 6:**
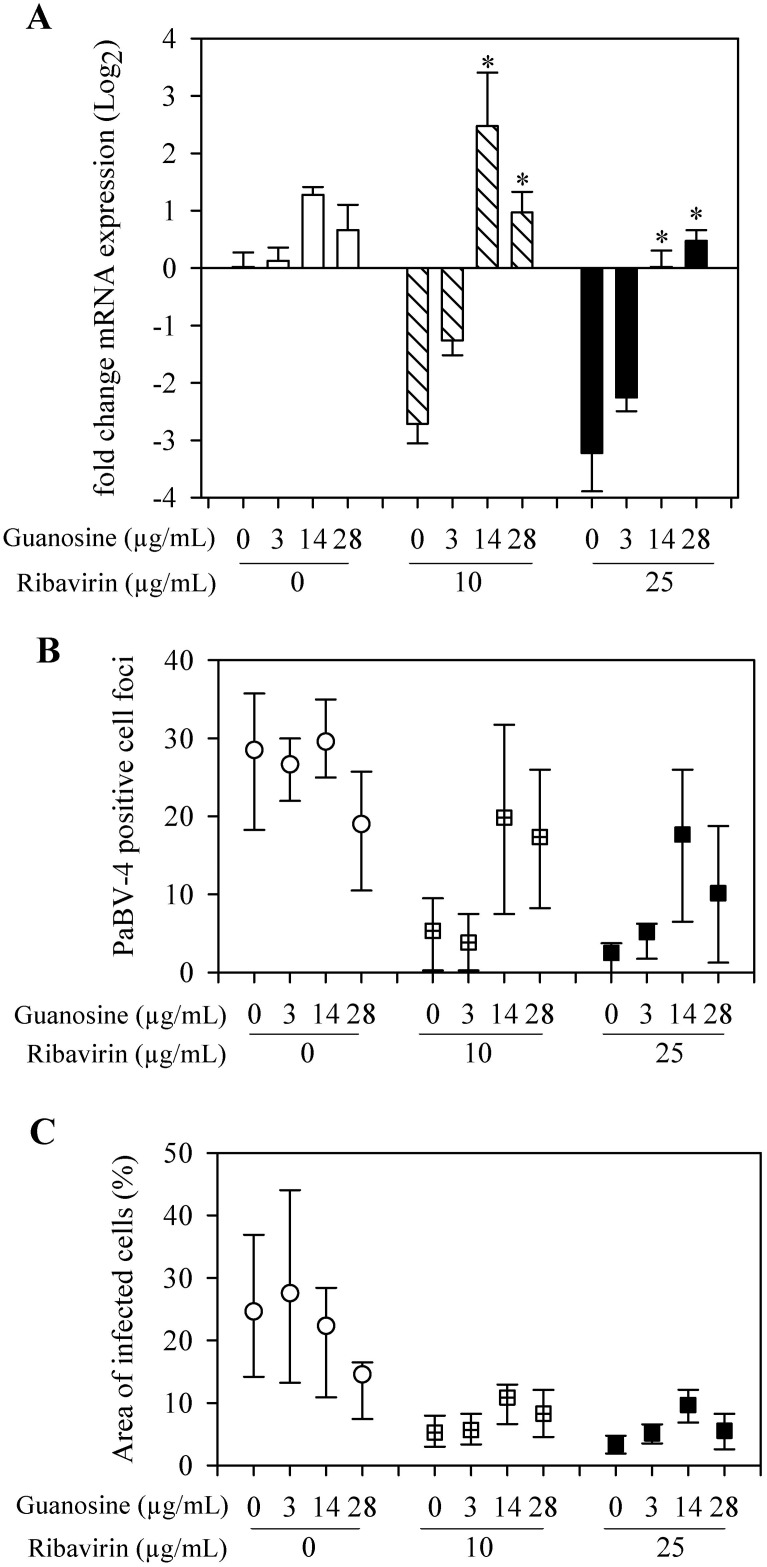
Guanosine inhibition of ribavirin antiviral activity on PaBV-4. (A) qRT-PCR results reported as mean (±SD) relative change in P gene mRNA expression, compared to control group (neither guanosine nor ribavirin added). *, compared with no guanosine within ribavirin treatment group (*P* <0.05, ANOVA with Holm-Sidak method). (B) Indirect immunocytochemistry results presented as median ± 25^th^ and 75^th^ percentiles positive stained cell foci. (C) Indirect immunocytochemistry results presented as median ± 25^th^ and 75^th^ percentiles of the percentage area of infected cells.

To test whether depletion of GTP pools was a mechanism of action contributing to the antiviral activity of ribavirin, 3 to 28 μg/mL guanosine was added to infected cells treated with ribavirin. The addition of guanosine reduced the antiviral activity of ribavirin in a dose-dependent manner. The relative expression of the P gene mRNA was significantly (P≤ 0.05) increased, between 3 to 5 fold increase, with the addition of 14 and 28 μg/mL of guanosine when compared to when ribavirin was present without guanosine (Fig 6A).

Indirect immunocytochemistry analysis of infected cells for N protein yielded similar results with numbers of PaBV-4 positive cell foci and area of infected cells increasing with 14 and 28 μg/mL guanosine (Fig 6B & 6C).

### Baicalein

**Fig 7:**
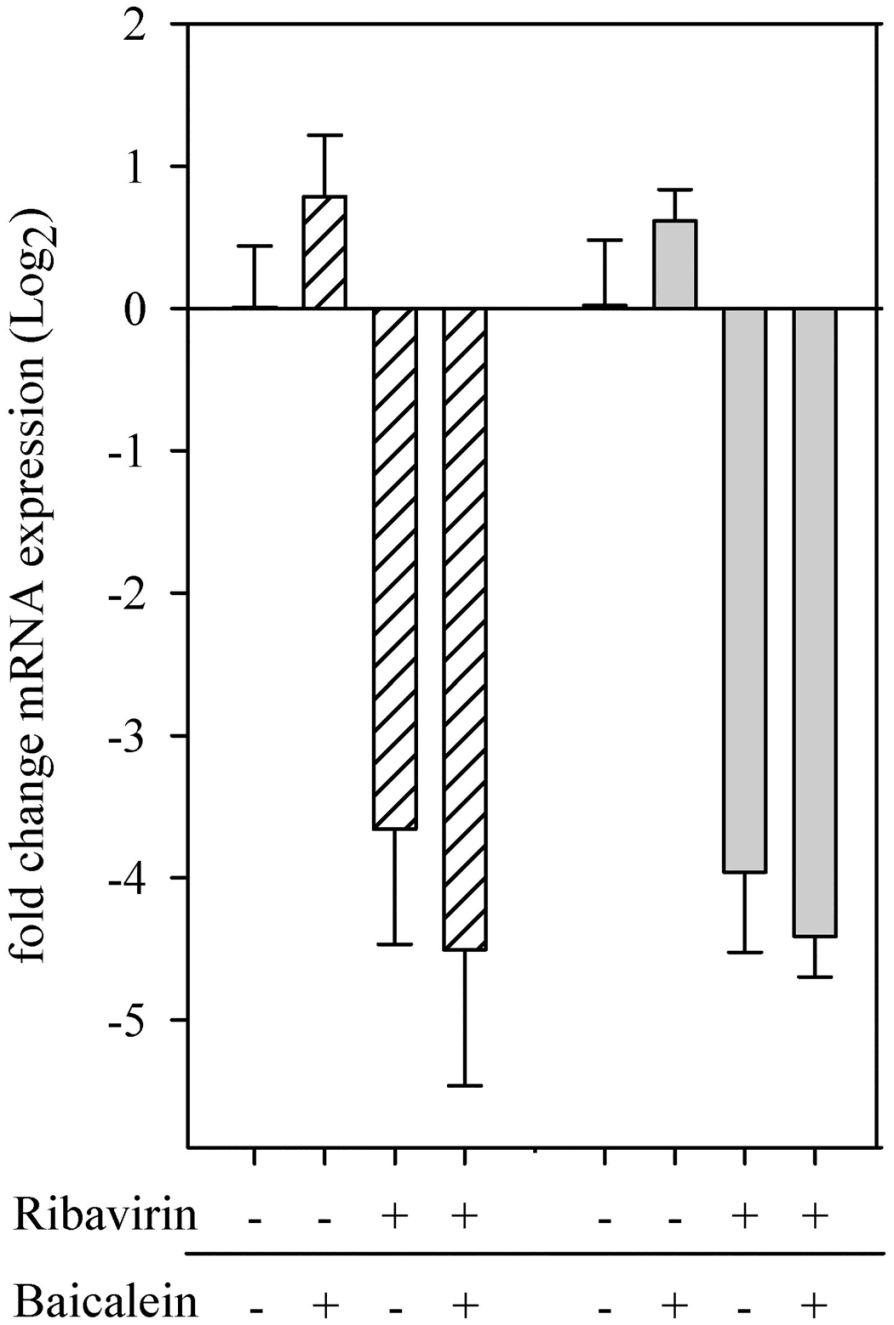
Potential anti-viral synergism of baicalein and ribavirin on PaBV-4. Duck embryonic fibroblasts infected with 7 (white hashed bars) or 64 (grey bars) ffu PaBV-4, incubated with 0.05 μg/mL baicalein, 5.0 μg/mL ribavirin, both drugs or neither. Cells were analyzed by qRT-PCR with results (mean ±SD) shown as the fold change in P gene mRNA expression relative to the expression of the groups treated with neither drug.

The effect of baicalein on the antiviral activity of ribavirin was investigated by comparing the expression of P gene mRNA with and without treatments. With the addition of 5.0 μg/ml ribavirin or with the combination treatment of ribavirin and baicalein, there was a 3.5 to 4.5 fold reduction in mRNA expression as compared with no treatment control, however combination treatment did not show mRNA decreasing more than ribavirin alone (Fig 7).

## Discussion

In this study we demonstrated that ribavirin, at concentrations between 2.5 and 25 μg/mL, had a dose-dependent inhibition on PaBV-4 replication as demonstrated by decreasing viral mRNA and viral protein loads in infected DEF cells. This decrease was demonstrated using two analytical methods that evaluated different products of viral replication, indirect immunocytochemistry assay for viral N protein and qRT-PCR for viral P gene mRNA expression. Viral protein synthesis rapidly declined with ribavirin treatment; after 24 hours of incubation with 25 μg/mL ribavirin, numbers of PaBV-4 infected cell foci and area of infected cells dropped to 2 and 9%, respectively, of the levels in untreated infected cells. This most probably represents background level of these assays, because similar values for infected cell foci and area of infected cells were also obtained from non-infected control cells (Fig 5A and 5B). Additionally, following 5 day exposure to 2.5 to 25 μg/mL ribavirin, there was between a 3 and 6 fold reduction in viral mRNA expression (Figs 4 and 6A) and viral mRNA levels did not recover to control levels till up to14 days after ribavirin removal (Fig 4). Our findings are comparable to studies on BoDV-1, a closely related bornavirus, where ribavirin inhibited transcription and reduced viral load in vitro [27,28]. Our study used only PaBV-4 in DEF cells, but it would be interesting to examine the susceptibility of other members of the species *Psittaciform 1 bornavirus* in additional cells lines, as most members of the family *Bornaviridae* exhibit cell specific tropism and growth characteristics [10] and different patterns of replication in various cell lines [34] and the efficacy of ribavirin treatment shows virus genotype and cell line variability [35–37].

The study was were carried out with DEF cells; PABV-4 replicates well in DEF cells and is not cytopathic [4,9]. The cytotoxic effect of ribavirin on DEF cells is unknown and varies with cell lines [23,24,28,38–40]. An additional variable is that commercially available preparations have compounding chemicals that may be toxic to cells in culture. Cytotoxic assays in our studied showed that the pure ribavirin and the commercial ribavirin product affected cell viability at concentrations above 720 μg/mL, well below the studied concentrations of 2.5 to 25 μg/mL (Fig 2).

Better understanding of the mechanism by which ribavirin inhibits PaBV-4 replication is an important step in its use in infected animals and will help elucidate the mechanisms of *Bornaviridae* infection and biology. There are a few excellent reviews on the proposed antiviral mechanism of action for ribavirin [17–19,41–44]. Proposed mechanisms of action for ribavirin’s antiviral activity include: (i) inhibition of inosine monophosphate dehydrogenase (IMPDH) resulting in the depletion of intracellular guanosine nucleotides (GMP, GDP, and GTP) pools; (ii) inhibition of viral RNA-dependent RNA-polymerase, RNA capping, and RNA synthesis; (iii) lethal mutagenesis due to misincorporation of nucleotides; and, (iv) immunomodulatory effects on Th1 and Th2 T lymphocytes. The mechanism by which ribavirin inhibits PaBV-4 replication is unknown. Our studies corroborate findings that one mechanism of action in ribavirin’s antiviral activity on *Bornaviridae* is likely to be the inhibition of IMPDH causing disruption in GTP required processes [28]. Though this is a proposed mechanism for ribavirin in many viruses, it has been shown not to be a mechanism against Influenza A [45], Lassa virus [46] and infectious salmon anemia virus [23]. Biologically, the implications of this mechanism of action in the clinical patient is still questionable, for in the animal, intracellular guanosine nucleotides pools are highly regulated and fine-tuned to provide for the cellular GTP needs [17].

Other mechanisms of action, such as mutated RNA, due to the misincorportation of cytidine and uridine instead of guanine or adenine, may factor in to ribavirin’s antiviral activity [23]. However, our study was not designed to investigate lethal mutagenesis or inhibition of viral RNA-dependent RNA-polymerase, RNA capping, and RNA synthesis as mechanism of actions. Additionally, it could not evaluate the immunomodulatory effects of ribavirin. Immunomodulation and the amelioration of the inflammatory response *in-vivo* may be an important aspect in ribavirin’s clinical application and efficacy [47]. An inflammatory reaction in infected neurons is seen in clinical cases of PaBV [48], as well as being part of the pathology in BoDV-1 [49]. Further studies on the mechanisms of action of ribavirin would be useful.

In investigating potential synergism of baicalein and ribavirin, the combination of drugs at a 1:100 ratio of Baicalein/Ribavirin did not appreciably increase the antiviral activity of ribavirin, there was less than a 1-fold reduction of P gene mRNA expression between ribavirin and ribavirin with the addition of baicalein. This is not consistent with the dose-dependent reduction of influenza A viral matrix protein gene expression seen by Chen et al [26]. These differing results may be due to baicalein’ s mechanism of action and PaBV-4 replication/infection cycle as compared to influenza. The mechanism of action for baicalein’ s antiviral activity is not well elucidated but is reported to be through blocking virus binding and entry into the cell [50–52] and inhibition of the enzymatic activity of reverse transcriptase [53]. Bornaviruses enter the cell through a receptor-mediated endocytosis and pH dependent fusion [54], and spreads mainly through a cell-to-cell transfer between contiguous cells [55], possibly in the form of a ribonucleoprotein complex [56]; this has yet to be fully elucidated for PABV. The specific mechanisms for the lack of individual or synergetic antiviral activity of baicalein on PaBV-4 were outside the scope of this project. But a method procedural difference between the two studies should be noted; whereas Chen et al. infected and treated cells simultaneously, we allowed a 24-hour incubation of DEF cells with PaBV-4 prior to any antiviral treatment. Our procedure was to simulate that viral infection would have occurred prior to ribavirin treatment in naturally infected animals.

## Conclusion

To our knowledge, this is the first report showing that the ribavirin significantly inhibits PaBV-4 replication in cell culture. We presume that the inhibitory effect on PaBV-4 replication may be mediated by IMPDH inhibition, but other mechanism such as lethal mutagenesis and inhibition on viral RNA-dependent RNA-polymerase, RNA capping, and RNA synthesis may be factors. These findings, and ribavirin’s immunomodulation properties, suggest that further studies on ribavirin’s pharmacokinetics, toxicity, and treatment efficacy against PaBV 1–7 in naturally and experimentally infected birds should be investigated.

## Supporting Information

S1 Dataset Relevant study raw data. (XLSX)
